# Reply to Uzoigwe: Modeling and the historical record

**DOI:** 10.1073/pnas.2417598121

**Published:** 2024-10-31

**Authors:** Elizabeth N. Blackmore, James O. Lloyd-Smith

**Affiliations:** ^a^Department of Ecology and Evolutionary Biology, University of California, Los Angeles, CA 90095; ^b^Department of Ecology and Evolutionary Biology, Yale University, New Haven, CT 06520

We thank the reader for their provocative response to our work (1, 2). However, the core premise that our findings are “in conflict with some compelling historical evidence” misinterprets our results and places too much stock in historical claims that experts no longer consider credible.

Regarding measles and smallpox, 16th-century trans-Atlantic transfers are well documented (3) and are entirely consistent with our results. As noted, sail voyages from Europe to the Americas during this period lasted roughly 35 to 70 d. Our analyses indicated that smallpox and measles introductions are very plausible on this timeframe; for example, in table 2, we estimate roughly a 25% risk of smallpox or measles transfer had the Santa María (1492) departed with one infected person on board. Supplementary analyses indicated that these results were robust across a broad range of conditions.

Regarding influenza, modern scholars widely view the hypothesis of a 1550s influenza outbreak in the Americas as uncertain (4, p. 270) and unsubstantiated (5, p.18; 6, p. 190). More generally, there is reason to be skeptical of influenza diagnoses based solely on written records. In contrast to smallpox and measles, influenza symptoms are nonspecific and can plausibly result from infection with a broad range of pathogens, including metapneumovirus, adenoviruses, and coronaviruses (7, 8). For this reason, the United States Centers for Disease Control require laboratory confirmation for definitive influenza diagnosis and use the more general term “influenza-like illness” to refer to cases based only on the classic symptoms of fever and sore throat (9). Notably, many pathogens causing “influenza-like” symptoms have significantly longer life cycles (10). Thus, written accounts of influenza-like illness during the 15th and 16th centuries do not, in themselves, contradict our assessment that early trans-Atlantic transfers of influenza were unlikely.

More generally, our framework was not presented as a definitive statement on global disease history. As we emphasized, pathogen introduction rates were highly sensitive to a range of factors which are not yet well characterized in historical systems. These include ship-board transmission rate, population susceptibility, pathogen natural history, and infection prevalence in ports of origin. Necessarily, we selected a small set of values for illustrative simulations. However, we do not exclude the possibility that different scenarios could have—and possibly did—occur, perhaps resulting from factors such as sexual immunity differences or epidemiologically meaningful pathogen evolution. For this reason, we explored a broader range of scenarios in supplementary analyses. In this initial analysis, we explicitly excluded zoonotic involvement for the sake of simplicity.

Theoretical disease modeling necessarily requires broad approximations of complex social and biological processes. Real-world comparisons offer a critical sense of whether our models can usefully approximate the biology of any given system. We consider model validation and parameterization to be a priority and in future work plan to assess this model’s predictions against relevant maritime records, such as ship medical logs. Currently, the historical record of past infectious disease outbreaks offers the best available comparison. On this score, we are satisfied that our predictions are consistent with the present-day historical consensus.

## References

[1] C. E. Uzoigwe, Christopher Columbus’ flu was different to ours. Proc. Natl. Acad. Sci. U.S.A. **121**, e2414921121 (2024).39480844 10.1073/pnas.2414921121

[2] E. N. Blackmore, J. O. Lloyd-Smith, Transoceanic pathogen transfer in the age of sail and steam. Proc. Natl. Acad. Sci. U.S.A. **121**, e2400425121 (2024). 39012818 10.1073/pnas.2400425121 PMC11287167

[3] J. R. McNeill, "Disease environments in the Caribbean to 1850" in Sea and Land: An Environmental History of the Caribbean, P. D. Morgan, J. R. McNeill, M. Mulcahy, S. B. Schwartz, Eds. (Oxford University Press, 2022), pp. 130–186.

[4] K. Harper, Plagues upon the Earth: Disease and the Course of Human History (Princeton University Press, 2021).

[5] D. R. Snow, K. M. Lanphear, European contact and Indian depopulation in the Northeast: The timing of the first epidemics. Ethnohistory **35**, 15–33 (1988).

[6] J. K. Taubenberger, D. M. Morens, Pandemic influenza—Including a risk assessment of H5N1. Rev. Sci. Tech. **28**, 187–202 (2009). 19618626 10.20506/rst.28.1.1879 PMC2720801

[7] A. Fowlkes , Viruses associated with acute respiratory infections and influenza-like illness among outpatients from the influenza incidence surveillance project, 2010–2011. J. Infect. Dis. **209**, 1715–1725 (2014). 24338352 10.1093/infdis/jit806 PMC5749912

[8] F. Krammer , Influenza. Nat. Rev. Dis. Primers **4**, 3 (2018). 29955068 10.1038/s41572-018-0002-y PMC7097467

[9] A. Budd , Manual for the Surveillance of Vaccine-Preventable Diseases. Chapter 6: Influenza. https://www.cdc.gov/surv-manual/php/table-of-contents/chapter-6-influenza.html. Accessed 17 September 2024.

[10] J. Lessler , Incubation periods of acute respiratory viral infections: A systematic review. Lancet Infect. Dis. **9**, 291–300 (2009). 19393959 10.1016/S1473-3099(09)70069-6 PMC4327893

